# Cerebral sinus venous thrombosis in a child with nephrotic syndrome

**Published:** 2014-10

**Authors:** Mahmood D. Al-Mendalawi

**Affiliations:** *Department of Pediatrics, Al-Kindy College of Medicine, Baghdad University, Baghdad, Iraq*

***To the Editor***

I have 2 comments on the interesting case report by Al-Rumayyan.^1^

First, though nephrotic syndrome is closely linked to the development of venous thromboembolism (VTE), concomitant inherited abnormalities of coagulation should be always considered. Common causes of hypercoagulability, also known as thrombophilia, include factor V Leiden, the prothrombin gene mutation, antiphospholipid antibodies, and hyperhomocysteinemia. The presence of genetic thrombophilia markers as factor V Leiden, prothrombin 20210A mutation, and antiphospholipid antibodies were found to significantly increase the patient’s risk of a thrombotic event. The relative risk of thrombosis in factor V heterozygotes was estimated to be at least 3 times higher than in the general population, whereas the increased risk of thrombosis in homozygotes was estimated to be 50 to 80 fold greater than those without the defect. Thromboembolic events were reported in approximately one third of antiphospholipid-positive patients. Other markers such as hyperhomocysteinemia and deficiencies of antithrombin, protein C, or protein S, when combined with the previous mutations, were noticed to significantly increase the patient’s risk of a thrombotic event.^2^ Al-Rumayyan^1^ solely relied upon normal genetic study for factor V Leiden mutation in the prothrombin gene and unremarkable family history with regards to coagulopathy to exclude concomitant thrombophilia in the studied nephrotic patient. I presume that Al-Rumayyan^1^ did not follow the most cost-effective approach in the studied patient, which includes initially screening for factor V Leiden, the prothrombin gene mutation, hyperhomocysteinemia, and antiphospholipid antibodies as these are the most common defects causing thrombophilia.^3^ Moreover, the negative personal and family history of VTE is not sufficient to exclude thrombophilia, and patients with VTE should be tested for inherited thrombophilia regardless of the patient’s past personal and family history for VTE.^4^

Second, the success of Al-Rumayyan^1^ to treat cerebral venous sinus thrombosis (CVST) in the studied patient using local tissue plasminogen activator is really pleasing. Other modalities of endovascular thrombolytic therapy including, balloon angioplasty, microguidewire and catheter disruption, and thromboaspiration using the Penumbra device have been recently evaluated in patients with CVST and showed promising results. Accordingly, they might have a role in the treatment of CVST in children when medical therapy fails and the patient is in a poor clinical condition.^5^

***Reply from the Author***

We sincerely appreciate the useful comments by Prof. Al-Mendalawi. His special highlights on genetic risk factors and medical and surgical management approaches of hypercoagulability (thrombophilia) in general are well taken. However, it might be questionable whether or not similar potential genetic risk factors were operating in our case. Notably, each pediatric patient with nephrotic syndrome complicated by intracranial venous sinus thrombosis is unique in etiological dimension, symptoms presentation, treatment response, prognosis, and neurological outcome. Probably, each patient with a specific disease developing intracranial venous sinus thrombosis is unlikely to have similar etiological denominators because there are a variety of genetic and environmental determinants of this complication. Nephrotic syndrome in children,^6^-^13^ and adults^14^ is an unequivocal risk factor for the development of CSVT, a rare but potentially dangerous complication. Additional risk factors of CSVT in children with nephrotic syndrome include thrombocytosis associated with iron deficiency anemia, diuretic therapy, and steroid treatment. Furthermore, clinical wisdom suggests that when medical interventions fail in the early management of CSVT, surgical approaches need to be used to save the life of children with nephrotic syndrome accompanied by life threatening CSVT.

***Ahmed R. Al-Rumayyan***

*College of Medicine, King Saud Bin Abdulaziz University for Health Sciences, Riyadh, Kingdom of Saudi Arabia*
